# Salvage liver transplantation after resection of colorectal cancer liver metastasis with favorable outcomes: a case report and review of the literature

**DOI:** 10.1186/s12876-021-01778-6

**Published:** 2021-04-27

**Authors:** Mahmoud Tabbal, Abdullah Mahmoud Alkhalifa, Abdullah Saleh AlQattan, Mohammed AlJawad, Mansour Ahmed Tawfeeq, Mohammed Saad Al Qahtani

**Affiliations:** 1 Hepatobiliary and Transplant Surgery, King Fahad Specialist Hospital – Dammam, Dammam, Saudi Arabia; 2 General Surgery Department, King Fahad Hospital of the University, Khobar, Saudi Arabia

**Keywords:** Colorectal liver metastasis, Liver transplant, Overall survival

## Abstract

**Background:**

Approximately 50% of patients with colorectal cancer (CRC) develop metastases most commonly in the liver. Liver transplantation (LT) can be used in certain cases of primary liver malignancy or in metastatic diseases, such as Neuroendocrine tumors. However, there are controversies regarding LT as a treatment option for liver metastasis from CRC due to poor outcomes in previously reported cases.

**Case presentation:**

We report a 37-year-old male who underwent resection of the left-sided colon due to cancer and was found to have synchronous liver metastasis for which he received chemotherapy. Later, he underwent a right hepatectomy, which was complicated by insufficient liver remnant function despite the preserved liver perfusion. Therefore, salvage liver transplantation was performed successfully with a good long-term outcome.

**Conclusions:**

Many studies examined the survival and quality of life in patients undergoing liver transplantation for unresectable colorectal liver metastasis; these studies include the SECA Study (secondary cancer) and others with favorable outcomes. We reviewed the literature and compared the outcomes of some of these studies in this article. Our case emphasizes that liver transplantation could be an option for some colon cancer liver metastasis (CLM) patients, specifically, as a salvage procedure. Thus, more research is needed to develop selection criteria for patients who may benefit from liver transplantation.

## Background

Colorectal cancer (CRC) is the third most common cancer worldwide, with more than one million cases diagnosed each year. Approximately 50% of patients with CRC develop metastases during the course of the disease, with the liver being the most common organ involved [1]. Treatment options for colorectal liver metastasis (CRLM) are hepatectomy, chemotherapy, ablation, embolization or hepatic artery infusion, whereas unresectable CRLM is generally treated with palliative chemotherapy [2]. Liver transplantation (LT) has been used as a treatment option not only in non-malignancy-related liver failure but also in certain hepatic malignancies, such as hepatocellular carcinoma (HCC) and hilar cholangiocarcinoma, or even in certain metastatic lesions, such as neuroendocrine tumors [1]. However, there are still controversies regarding LT as a treatment option for unresectable CRLM due to the poor outcomes in previously reported cases [1, 2]. However, with the recent advancements in colorectal cancer staging, chemotherapy, and immunomodulating drugs, LT has been reintroduced as a possible option for patients with unresectable CRLM, liver failure secondary to liver resection, or in cases of disease recurrence after hepatectomy [2]. We report a case of colon cancer and liver metastasis in a patient who was treated with resection of the primary cancer followed by a right hepatectomy that was complicated by acute liver failure and followed by a salvage LT procedure. This case is reported in accordance with the CARE reporting checklist.

## Case presentation

We report a case of a 37-year-old male who was diagnosed with left-sided colon cancer in 2015 and underwent resection of the primary cancer with a protective loop ileostomy in another hospital followed by a stoma reversion in January 2016. Primary tumor pathology showed a 5 cm moderately differentiated adenocarcinoma (T3N1bMx, KRAS wild type, MSS/MSI-L intact protein) in the splenic flexure. Staging computed tomography (CT) of the chest, abdomen, and pelvis (CAP) was performed and showed 3 liver lesions with elevated blood levels of carcinoembryonic antigen (CEA) of 8 ng/ml. Therefore, the patient underwent chemotherapy and was started on oxaliplatin, capecitabine, and bevacizumab. The CT CAP was repeated after completing the course of treatment and showed that he had some response to chemotherapy. The patient was then kept on maintenance chemotherapy (bevacizumab & Xeloda) and referred to our hospital for possible further management after completing a total of 12 cycles of therapy.

**Fig. 1:**
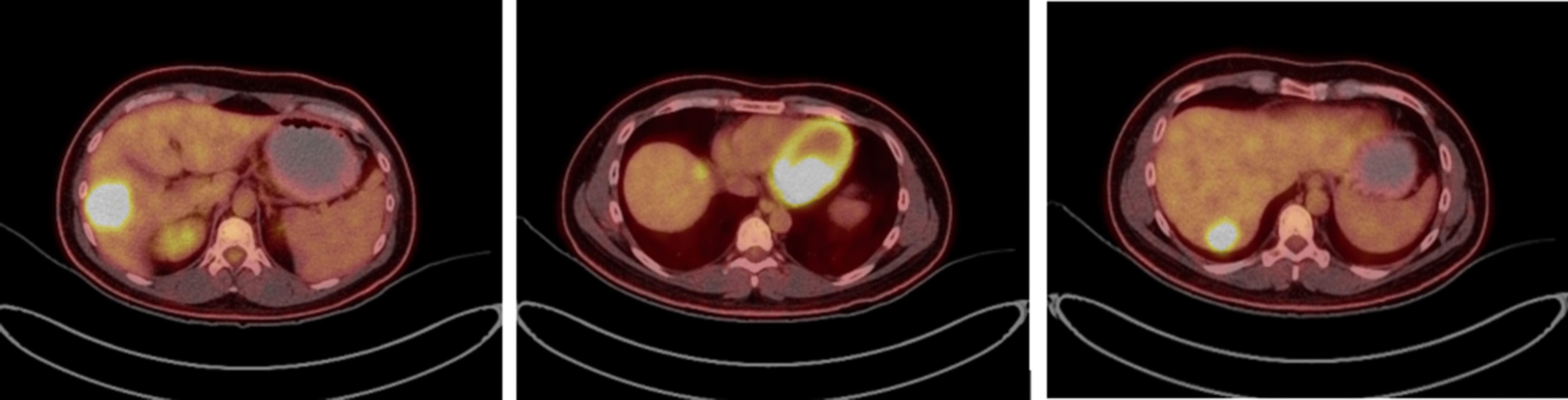
PET/CT scan showing the metastatic liver lesions

**Fig. 2:**
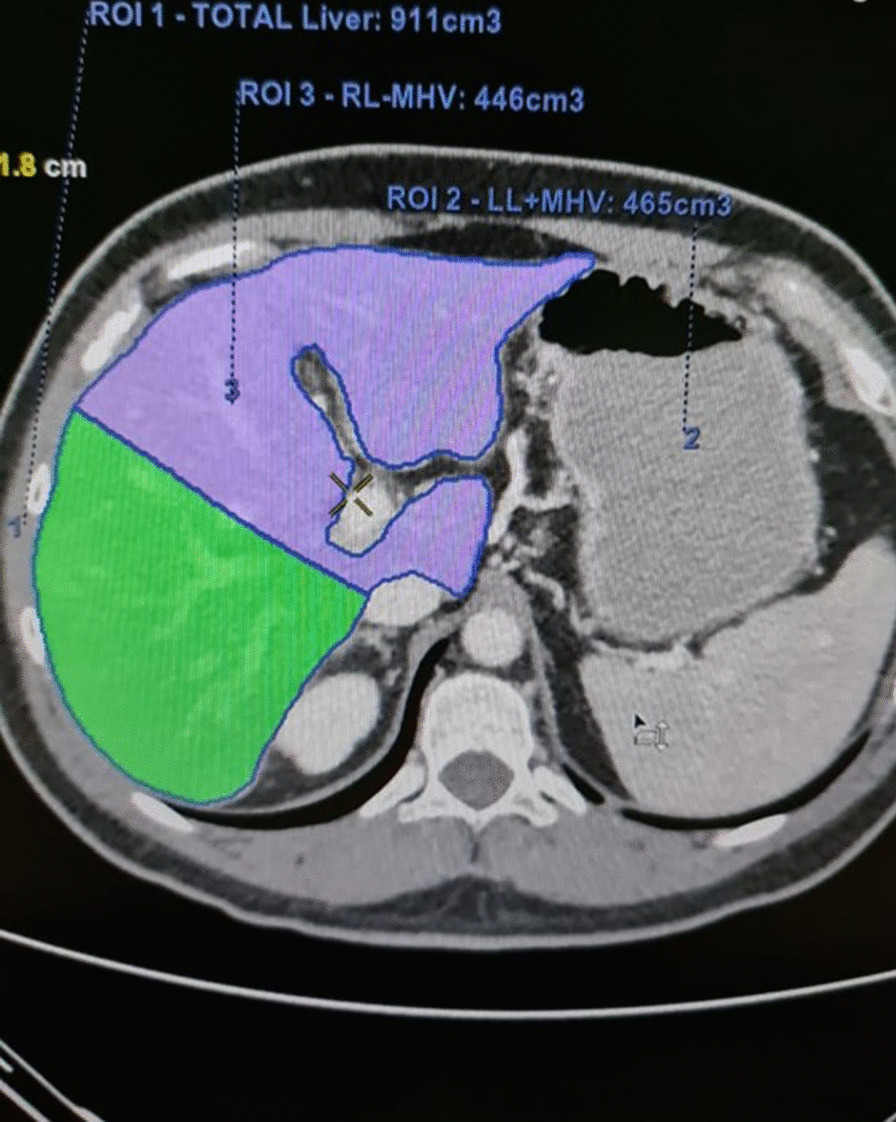
CT of the abdomen with volumetry showing an estimated volume of the possible future liver remnant (left liver lobe) of approximately 51%

In our hospital, the patient was re-evaluated with a CT CAP and hepatic magnetic resonance imaging (MRI). The liver MRI showed a lesion in segment 7 posteriorly, measuring 1.5 × 2.1 cm, a lesion in segment 6, measuring 3 × 3 cm, and another suspicious small lesion in segment 4A. These lesions were slightly larger than the lesions on previous images, and a positron emission tomography (PET) CT scan confirmed the marginal progression of the disease in the liver (Fig. 1). In October 2017, a multidisciplinary decision was made to proceed with the parenchyma-preserving wedge resection with the possibility for a major resection if necessary. Preoperative CT of the abdomen with volumetry showed an estimated volume of the possible future liver remnant (left liver lobe) of approximately 51% (Fig. 2). Unfortunately, an intraoperative ultrasound showed the largest lesion with close proximity to the main right portal vein.

Knowing that the preoperative liver function test was normal and the future liver remnant by the CT volumetry was more than 50%, to avoid having a positive margin by preserving the right portal vein, the decision was made to proceed with the right hepatectomy.

**Fig. 3:**
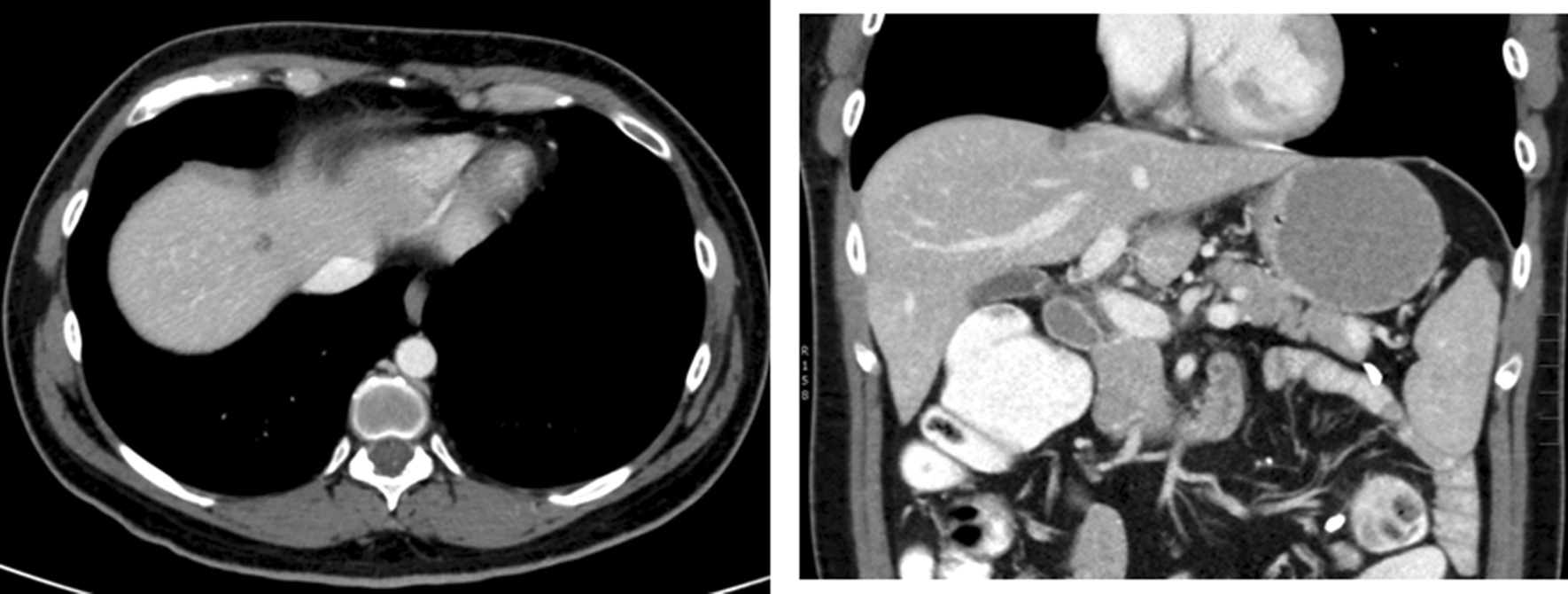
CT of the abdomen showing segment 4A/8 liver lesion

The lesion in segment 4A was tiny, very close to segment 8 and away from the middle hepatic vein (Fig. 3). Therefore, it was included in the resection of the right hepatic lobe without concern of compromising the blood supply or drainage of the left hepatic lobe, and the parenchymal resection of the segment 4A was limited.

**Fig. 4:**
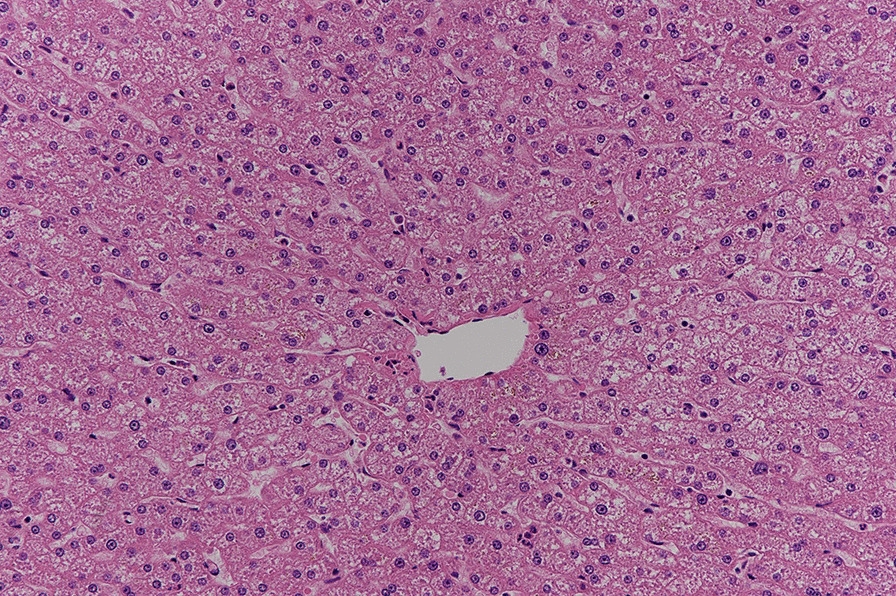
Microscopic image of the right liver lobe showing normal background liver tissue with no element of necrosis (20×)

The quality of the remnant liver was grossly marginal, possibly due to extensive preoperative chemotherapy. Intraoperative ultrasound showed good perfusion of the remaining left lobe, with the normal flow in the left hepatic artery, left hepatic vein, middle hepatic vein, and left portal vein. Pathology of the resected right lobe showed 3 metastatic nodules ranging between 1.2 and 4 cm, with negative resection margins of at least 1.5 cm. The background liver of the resected right lobe was normal (Fig. 4).

Postoperatively, the patient was acidotic with a high lactate level of 13 and an international normalized ratio (INR) of 3. He was kept intubated with inotropic support. Liver Doppler ultrasound was performed again and showed adequate blood flow in the remnant liver. However, the patient’s liver function continued to deteriorate, and he became more encephalopathic but with repeatedly normal liver Doppler ultrasound demonstrating the patency of the vasculature on days 1, 2, and 6 postoperatively.

**Fig. 5:**
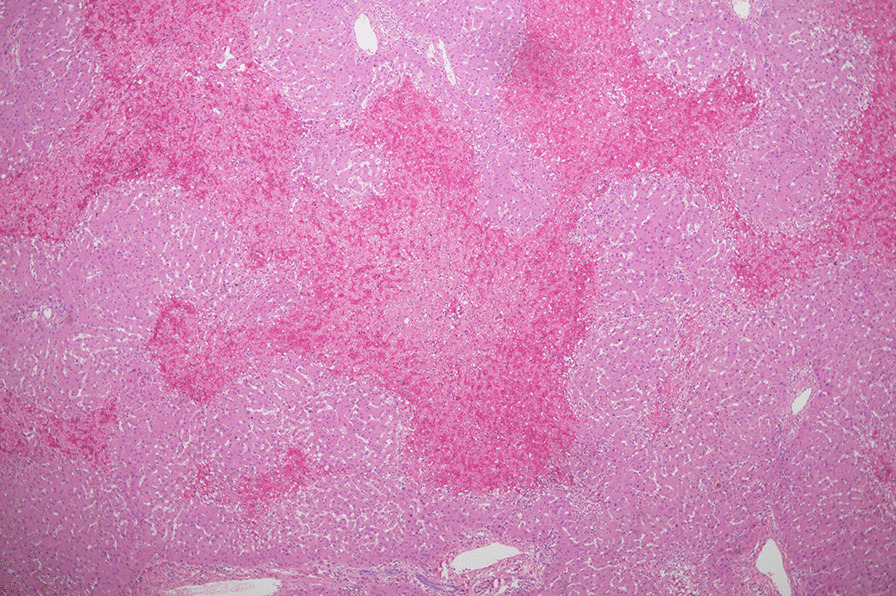
Microscopic image of the explanted liver showing extensive necrosis (4×)

The case was discussed with the transplant team to consider liver transplantation at this stage. Given that this patient was young, we decided to proceed with the salvage LT. The patient underwent deceased donor orthotopic liver transplantation in November 2017. The pathology of the explanted liver showed extensive necrosis (Fig. 5). The patient received induction immunosuppression (IS) therapy with methylprednisolone and then maintenance IS with steroids, tacrolimus, and mycophenolate. Mycophenolate was replaced by sirolimus two months post-transplantation for its anti-proliferative effect. The patient recovered well and was discharged home one month after the transplantation in good general condition. He had 2 episodes of mild acute cellular rejection, and the last episode was more than 12 months ago, both of which resolved completely with low-dose pulse steroids. His most recent follow-up (40 months after the transplantation) showed normal liver function, normal CEA of 1.6 ng/ml and the CT CAP showed no evidence of recurrence or metastasis.

## Discussion and conclusion

Liver transplantation has good 5-year overall survival (OS) in patients with an HCC of more than 80%. Le Treut et al. reported a 5-year OS of 52% and a disease-free survival of 30% from 213 patients who were treated with LT for metastatic liver neuroendocrine tumors [3]. Before 2000, multiple studies on LT for unresectable CRLM were conducted and yielded poor outcomes [4]. However, LT for unresectable liver metastases has recently gained interest again due to the advancements in cancer staging, chemotherapy, and immunomodulating drugs [2].

Liver resection is the only curative treatment option in patients with CRLM, achieving a 5-year OS of more than 25%. However, more than 75% of these patients present with unresectable liver metastasis. Some onco-surgical strategies have been employed, but more than 60% of them are unsuitable for complete resection. Unfortunately, the only treatment for these patients is palliative chemotherapy, which achieves a 5-year OS of less than 10% [5].

A Norwegian randomized clinical trial on SECA-I (SEcondary CAncer) that started in 2006 to assess the survival and quality of life of patients undergoing LT for unresectable CRLM showed an estimated 5-year OS of 60% with a better quality of life [5]. Additionally, Toso et al. published the results from a multi-institutional retrospective cohort study involving 12 patients with the CRLM who were treated with the LT with similar results to the SECA study, with a 5-year OS of 50% ± 16% [6]. Interestingly, two patients with the unresectable CRLM had no recurrence 9 and 21 years after the LT [7].

Dueland et al. conducted a study comparing patients with unresectable CRLM who underwent LT (SECA-I trial) with those who received chemotherapy (NORDIC VII trial), which showed a significant difference in OS in favor of LT [8]. Moreover, Dueland et al. compared the results of patients who underwent LT for unresectable CLRM and those who underwent LT for HCC. They observed that despite the higher tumor load in a selected number of patients with the CLRM, the 5-year survival rate was better than or similar to that of patients with HCC [9]. Smedman et al. studied the tolerability, safety, and efficacy of chemotherapy given for 23 patients with CRLM who underwent LT after they developed relapse, concluding that it was safe and well-tolerated, with no associated graft loss [10].

Currently, there are several ongoing clinical trials that might confirm these results, including the TRANSMET trial (NCT02597348), in which they recruited patients with unresectable CRLM with either LT plus neoadjuvant chemotherapy or chemotherapy alone, and the SECA-III trial (NCT03494946) comparing LT to the best multimodal alternative treatment (chemotherapy ± locoregional therapies). Finally, a clinical trial led by the University Health Network in Toronto, Canada (NCT02864485) assessed the safety and effectiveness of neoadjuvant chemotherapy followed by living donor LT [11].

The data of the SECA-I study indicate that LT can offer selected patients with unresectable CLRM better survival and excellent quality of life when compared to previous outcomes. Unfortunately, the recurrence rate is still high and requires better selection criteria and perioperative treatment [12]. Hagness et al. reported that 19 out of 21 patients enrolled in the SECA study developed disease recurrence. The median time of recurrence was 6 months (2–24 months). The most frequent site was the lung [13]. Interestingly, Grue et al. compared the growth of pulmonary metastatic lesions following LT in patients receiving immunosuppressive therapy and in patients who did not receive immunosuppressive therapy and concluded that immunosuppressive drugs did not accelerate the growth of the pulmonary metastatic lesions [14].

Hagness et al. reported some factors associated with better outcomes: time from primary cancer surgery to LT of more than 2 years, stable disease or partial response to chemotherapy at the time of LT, CEA level less than 80 µg/L before LT, and diameter of the tumor of less than 5.5 cm [12]. Grue et al. analyzed the data from the SECA study and predicted other factors associated with better outcomes, including the pre-transplantation metabolic tumor volume and the total lesion glycolysis in the liver metastasis from the PET/CT scan [14].

The authors of the SECA-I trial conducted another clinical trial, “SECA-II’’, with stricter inclusion criteria. The patients in SECA-II in comparison to SECA-I had lower numbers of metastatic lesions, size of the largest liver lesions, CEA levels, Fong Clinical Risk Score (FCRS), and Oslo Score. Additionally, none of the patients on chemotherapy had progression of the disease or the CEA levels > 80 µg/L pre-LT. These results affirm the association of these factors with a better prognosis, as the estimated 5-year OS was 83%, and 4 patients had no relapse in more than 2 years compared to the SECA-I study in which all 11 patients who underwent follow-up had a relapse [15]. The presented patient in this current report had many of these factors, which could explain the long survival without relapse.

In FCRS, 0–5 points were calculated giving 1 point for the following: synchronous metastatic disease (less than 12 months from diagnosis), lymph node-positive primary tumor, more than 1 lesion, a size larger than 5 cm, and CEA > 200 mg/L. The Oslo Score (0–4 points) was calculated by giving 1 point for each of the following pretransplant characteristics: largest lesion > 5.5 cm, plasma CEA levels > 80 mg/L, time from the surgery of primary tumor to LT of less than 2 years, and progressive disease on chemotherapy at the time of LT.

To the best of our knowledge, the data in the English literature regarding LT for patients with CLRM who developed postoperative liver failure (POLF) are scarce. In the retrospective study by Toso et al., 1 out of the 12 patients underwent LT for POLF and concluded that the patients with CLRM who underwent transplantation as upfront, salvage, and vena cava involvement had worse disease-free survival than the patients with planned LT [6]. However, good survival was reported in multiple case reports, such as in Hrehoret et al., who presented a case of unresectable CLRM who underwent LT due to subacute liver failure, which prolonged his survival and offered him a better quality of life [5]. Honori et al. reported a case study in which the patient survived more than 10 years after LT for POLF [16]. Uskudar et al. reported 2 cases who developed liver failure after liver resection followed by hepatic artery infusion chemotherapy with a survival of more than 5 years for the first case and 2 years for the other case [17]. This outcome was also evident in our case, with more than 32 months of disease-free survival time and overall survival.

One of the challenges to treat patients with CRLM with LT is that there is a shortage of deceased organ donors and using these grafts in these patients at the current time could negatively impact the other patients on the waiting list with conventional indications. However, currently, there are two trials recruiting patients with the hope of overcoming this issue: RAPID (Resection And Partial Liver segment 2/3 transplantation with Delayed total hepatectomy) and LIVER-T(W)O-HEAL. The former trial is where a two-stage hepatectomy is performed followed by transplantation of the left lateral lobe (segments 2 and 3) of the deceased graft. The other trial is one in which two-stage hepatectomy is performed followed by left lateral living donor LT [18, 19].

Our case emphasizes that LT could be an option for some patients even as a salvage option and can provide a better disease-free and overall survival rate and quality of life. Thus, more research is needed to identify the optimal selection criteria for patients who may benefit from LT, particularly in countries where there is a shortage of organ donors. However, our current case carries its own limitations in the form of a short interval of time between surgery of the primary tumor and the liver transplant, the acute setting in which the LT was performed, and underestimation of the quality of the background liver based on the gross appearance of the liver intraoperatively driving the decision for an upfront right hepatectomy. In such cases, a two-stage resection (associating liver partition and portal vein ligation for the staged hepatectomy (ALPPS)) with segment 4-A clearance and subsequent right hepatectomy might have been an option.

## Data Availability

Data sharing does not apply to this article, as no datasets were generated or analyzed during the current study.

## Abbreviations

CEA: Carcinoembryonic antigen
CAP: Chest Abdomen Pelvis
CRC: Colorectal cancer
CRLM: Colorectal liver metastasis
CT: Computed tomography
FCRS: Fong clinical risk score
GCS: Glasgow coma scale
ICU: Intensive care unit
INR: International normalized ratio
LT: Liver transplantation
MRI: Magnetic resonance imaging
OS: Overall survival
SECA: Secondary cancer
